# Comparison of anterior and posterior approaches for hip resurfacing arthroplasty: a gait analysis study

**DOI:** 10.1186/s13018-025-06457-w

**Published:** 2025-12-02

**Authors:** Milos Brkljac, Amy Maslivec, Natasha Allott, Kartik Logishetty, Justin Cobb

**Affiliations:** 1 MSk Lab, Imperial College London, London, UK; 2 University College London, London, UK; 3 University College London Hospitals NHS Foundation Trust, London, UK

**Keywords:** Anterior approach, Posterior approach, Hip resurfacing, Hip arthroplasty, Gait analysis

## Abstract

**Background:**

Hip resurfacing arthroplasty (HRA) is now only rarely performed, but usually using the posterior approach (POS), while total hip arthroplasty is now commonly performed using the direct anterior approach (DAA). This study aims to compare outcomes between these two approaches for HRA using gait analysis, the oxford hip score (OHS), metabolic equivalent of task (MET).

**Methods:**

Seventeen unilateral DAA and 17 POS HRA males were matched for age and BMI. Patients underwent instrumented treadmill gait analysis and completed patient reported outcome scores (PROMs) at a mean of 1.5 (0.9–1.8) years post-operatively. Kinematics and kinetics were recorded using motion capture and force plate data. Group differences were assessed using statistical parametric mapping. These data were compared to a group of 19 healthy male controls matched for age and BMI.

**Results:**

Gait analysis postoperatively revealed no significant differences in hip kinematics in either the coronal or sagittal planes between the posterior and direct anterior approaches. Statistical parametric mapping showed no differences in vertical ground reaction forces across the stance phase. Spatiotemporal gait parameters, including top walking speed, cadence, step length, and step width, were comparable between groups and closely aligned with healthy controls. Both cohorts achieved similar postoperative OHS (mean:48, *p* = 0.651) and MET scores (POS:13.1 vs DAA:12.6, *p* = 0.856).

**Conclusions:**

This is the first study to compare gait following HRA via both the POS and DAA. At one-year postoperatively, both approaches restored gait patterns comparable to healthy controls, with no significant differences in kinematics, kinetics, or spatiotemporal parameters. PROMs were similarly excellent across groups indicating high functional recovery and engagement in moderate-to-vigorous physical activity.

## Introduction

The functional demands on hip arthroplasty patients continue to rise. This trend reflects the procedure’s success and implant longevity, making it a viable option for individuals in their 40s and 50s [1]. Consequently, younger high-demand patients often opt for hip resurfacing due to its reported superiority in functional outcomes [2–4]. Currently available Metal-on-Metal (MoM) designs retain some controversy due to adverse reactions to metal wear debris. However, excellent results exist when performed well and using proven designs, such as the Birmingham Hip Resurfacing (BHR) [5]. More recently, promising early results [6] with ceramic-on-ceramic (CoC) bearings suggest a likely resurgence of interest in hip resurfacing. As hip resurfacing gains popularity, the surgical approach for this technically demanding procedure will face increasing scrutiny. Crucially, its impact on recovery and functional outcomes must be carefully evaluated.

Treadmill gait analysis provides an objective measure of functional performance, enabling quantitative assessment of walking at both normal and high speeds. This more sophisticated measure of function demonstrates symmetrical gait in healthy individuals, while osteoarthritic patients exhibit notable asymmetries. A systematic review reported that total hip arthroplasty (THA) improves gait symmetry, but fails to restore gait to normal in terms of walking speed, stride length or sagittal range of motion [7], while in a randomised trial HRA restored a more physiological gait at higher walking speeds using the posterior approach [8]. Both CoC HRA and the MoM BHR have been shown to restore gait parameters nearly indistinguishable from healthy controls [9, 10].

Normal walking is considered a prerequisite for more demanding activities, such as running, jumping, and sport [11, 12]. The success of hip arthroplasty is assessed in the NHS using a patient-reported outcome measures (PROM), the Oxford Hip Score, A modal score of 48/48 has been documented in randomised trials, limiting its value in high-functioning patients. More recently, the metabolic equivalent of task (MET) score has been proposed as an alternative, offering a patient-centred, normally distributed measure of postoperative physical activity intensity that better captures higher-level functional recovery linked to health and mortality risk [13].

The direct anterior approach to the hip has become the standard approach for total hip arthroplasty in much of the developed world [14], with good evidence in its favour in the short term [15], with objective evidence from gait analysis [16]. There are limited published studies describing HRA performed through this anterior approach [17–22]. The posterior approach (POS) is the approach used in most long term studies of hip resurfacing [23, 24].

With the resurgence of interest in hip resurfacing with alternative bearing couples, there is a mismatch between the direct anterior approach used by the majority of total hip arthroplasty surgeons and the posterior approach used by the majority of hip resurfacing surgeons. We surmised that the surgical approach used for hip resurfacing would not have any lasting impact on function.

### Objectives

The primary aim of this study was therefore to evaluate the impact of surgical approach on gait biomechanics in high-functioning patients one year after hip resurfacing arthroplasty, compared with a healthy control group. The secondary aim was to report any differences in metabolic equivalent of task (MET) scores across the two groups.

## Methods

### Study population

Thirty-four male patients who had received a unilateral MoM hip resurfacing (Birmingham Hip Resurfacing, Smith & Nephew) were selected for this study from a longitudinal study of gait (ethics reference number 14/NS/1045).

Patients had either a posterior (POS, n = 17) or a direct anterior (DAA, n = 17) approach to surgery by the same surgeon, who changed his approach to the direct anterior two years after converting to DAA for total hip arthroplasty.

Exclusion criteria included a BMI greater than 40, over 70 years old or having severe comorbidities. All patients required unilateral HRA, they had no osteoarthritis (OA) or other surgery of their contralateral limb and were able to complete a gait analysis assessment 12 months post-operatively. Nineteen healthy control volunteers (CON) were matched for age, sex, and BMI, if they had no evidence of hip or knee osteoarthritis, no history of hip or knee surgery or injury, or any other lower limb dysfunction (Table 1).

**Table 1:** Patient Demographics by Surgical Approach and Control Group

	POS (n = 17)	DAA (n = 17)	CON (n = 19)
Age (Years)	51 (8)	54 (10)	51 (13)
BMI (kg/m^2^)	25 (2)	26 (2)	26 (4)
Time postop (Years)	1.1 (0.2)	1.2 (0.2)	–

Data are presented as means (standard deviation, SD) for age, time from surgery to assessment and body mass index (BMI). POS, posterior approach; DAA, direct anterior approach; CON, control group

### Sensitivity analysis (a priori)

To contextualise the adequacy of our sample, we performed a G*Power-equivalent sensitivity calculation. With n = 17 per surgical group (two-tailed α = 0.05; power 1–β = 0.80), the detectable between-group effect for a two-sample comparison is Cohen’s d = 0.99 (large). For the three-group ANOVA (DAA, POS, CON; N = 53), the detectable omnibus effect is Cohen’s f = 0.45 (large). Using variance typical of approach-comparison THA gait at ≈1 year (cadence SD ≈ 2.5–3.0 steps·min⁻^1^; step-length SD ≈ 2.0 cm), this corresponds to minimal detectable differences of ≈ 2.5–3.0 steps·min⁻^1^ (cadence) or ~ 2.0 cm (step length).

### Patient reported outcome measures (PROMs)

The Oxford Hip Score (OHS), and Metabolic Equivalent of Task Score (MET) were completed post-operatively.

The OHS (range 0–48) assesses hip pain and function, with higher scores indicating better outcomes. The MET score was calculated using the same methodology to Edwards et al. [25].

### Gait protocol

All patients underwent treadmill gait analysis post-operatively (0.9–1.3 years) using an instrumented treadmill [10] (HP/COSMOS, Hab International). The vertical components of ground reaction forces (GRF) were collected on tandem force plates (1000 Hz). After 5 min of familiarisation walking at 3km/hr, the speed of the treadmill was increased to 4 km/hr then further increased by 0.5 km/hr every 60 s until the patient reached their self-determined top walking speed (TWS). Patients wore their own comfortable footwear and were secured in a safety harness throughout the analysis and were able to stop at any point. A 3D motion-capture system (200Hz, VICON Nexus, Oxford Metrics Ltd, Oxford, UK) with 34 anatomically placed body markers on the lower extremities and pelvis was used to record kinematic data [26].

### Data analyses

Data was collected during a 30 s period at each walking speed, and the average of 10 steps for each limb was used for further analysis. GRF was normalised to body weight and were time normalised to 100% of the stance phase of the gait cycle. For kinematic data, the 3D marker trajectories were collected at 100 Hz with a 10-camera 3D motion capture system and was time normalised to 100% of the gait cycle and processed in VICON nexus. All data was analysed using custom Matlab scripts (Version 2022a, The Mathworks Inc., United States).

### Spatiotemporal gait variables

Top walking speed (TWS), cadence, step length of the operated limb, and step width were recorded. TWS was determined as the fastest walking speed achieved before breaking out into a run or being limited by discomfort.

### Ground reaction force profile

Vertical GRFs profiles were collected during the stance phase of the gait cycle and reported at 6 km/hr as this was the highest common walking speed.

### Kinematic data

Hip angle in the sagittal and coronal plane was collected over the gait cycle. The angle values were ‘zeroed’ to 30 for sagittal, and 0 for coronal, to allow for a better comparison in range of motion, and do not therefore depict absolute hip angle [12].

### Statistical analysis

All data were assessed for normality using the Shapiro–Wilk test. Parametric tests were applied to normally distributed datasets, while non-parametric tests were used for non-normally distributed data. Between-group comparisons for the Oxford Hip Score (OHS) were conducted using the Mann–Whitney U test, while the MET score was compared using an independent samples t-test.

For spatiotemporal gait parameters, ground reaction forces (GRF), and joint kinematics, either one-way analysis of variance (ANOVA) or the Kruskal–Wallis test was used, depending on the distribution of each dataset. Normality of residuals was checked (Shapiro–Wilk; Q–Q plots) and homogeneity of variances with Levene’s test. Where Levene’s test indicated heteroscedasticity but group sizes were similar, ANOVA results were retained and interpreted with caution, as prespecified. For time-continuous data such as GRF and hip joint angles over the stance phase, statistical parametric mapping (SPM; spm1d) was employed. A one-way SPM{F} test was conducted across the normalised time series (0–100% of stance), identifying regions where the test statistic exceeded the critical threshold corresponding to *p* < 0.05; when present, post-hoc SPM{t} pairwise comparisons were performed with appropriate multiplicity control. These regions were considered statistically significant.

## Results

### Patient demographics

Patient demographics are presented in Table 1. The median age (IQR) was 51 (47–61) years in the POS group, 55 (47–60) years in the DAA group, and 42 (40–62) years in the Control group. A Kruskal–Wallis test showed no statistically significant difference in age between the groups (*p* = 0.517). Body mass index (BMI) was also comparable across groups, with mean values of 25 (POS), 26 (DAA), and 26 (controls), showing no significant difference using one-way ANOVA (*p* = 0.517). Similarly, the time from surgery to assessment did not differ significantly between the surgical groups, with a median of 1.1 years for POS and 1.2 years for DAA (Mann–Whitney U test, *p* = 0.061). No patients had undergone revision surgery or reoperation at the time of writing.

### Patient reported outcome measures

**Fig. 1:**
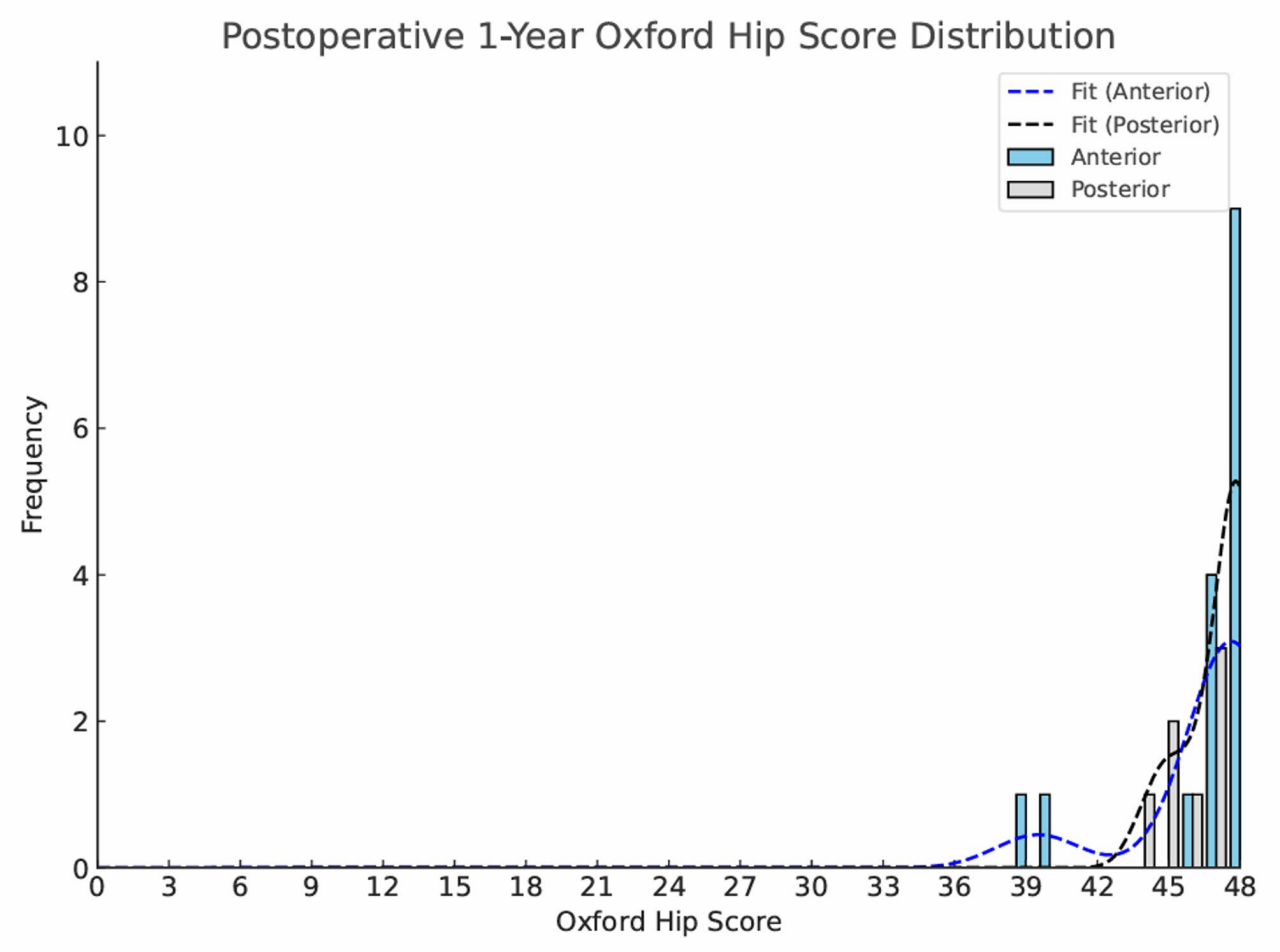
Histogram comparing 1-year postoperative Oxford Hip Scores between patients treated via anterior and posterior hip resurfacing approaches. Scores are plotted side-by-side. Both groups demonstrated excellent outcomes with a strong ceiling effect. Kernel density estimates (KDE) are overlaid to illustrate the distribution and skew of the data. Sample sizes: n = 14 (DAA); n = 17 (POS)

**Fig. 2:**
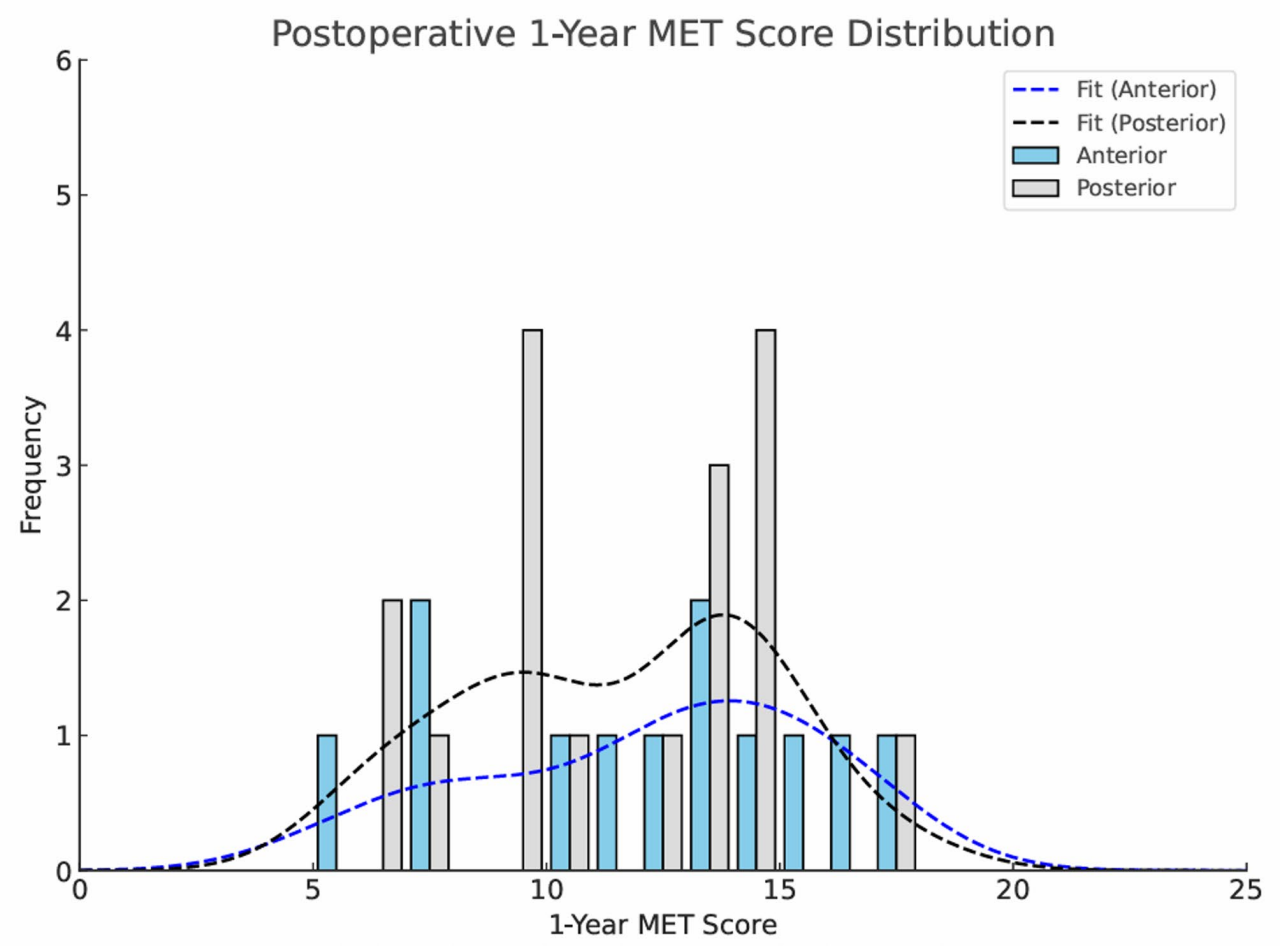
Histogram comparing postoperative MET scores between anterior and posterior hip resurfacing approaches. Both anterior and posterior groups demonstrated comparable distributions with a near-normal pattern, suggesting similar levels of postoperative physical activity across groups. Kernel density estimates (KDE) are overlaid to illustrate the symmetry and spread of the data

The postoperative scores are demonstrated in Figs. 1 and 2. No significant differences were observed between the postoperative scores for POS and DAA groups respectively for the Oxford Hip Score (OHS: 48 vs. 48, *p* = 0.651),), or MET score (13.1 vs. 12.6, *p* = 0.856).

### Spatiotemporal gait parameters

Patient walking characteristics are presented in Table 2. A one-way ANOVA revealed a significant difference between groups for top walking speed (TWS; F(2,48) = 5.55, *p* = 0.007). However, no significant differences were found for normalised top walking speed (TWS_norm; F(2,48) = 0.40, *p* = 0.673), step length (F(2,48) = 0.90, *p* = 0.414), cadence (F(2,48) = 1.53, *p* = 0.229), or step width (F(2,48) = 1.07, *p* = 0.349). Levene’s test confirmed homogeneity of variances for all variables except TWS (*p* = 0.007), and therefore the ANOVA result should be interpreted with caution.

**Table 2:** Walking characteristics by surgical approach and control group

	POS (n = 17)	DAA (n = 17)	CON (n = 19)
TWS (km/hr)	7.5 (6–8)	7.0 (6.5–8)	7.0 (6–8)
TWSnorm	0.66 (0.04)	0.65 (0.05)	0.63 (0.12)
Step length (cm)	46.5 (3.3)	46.5 (2.0)	47.3 (1.7)
Cadence	104 (5.8)	103 (4.8)	104 (3.8)
Step width	8.1 (2)	8.5 (2.1)	8.5 (1.2)

Data are presented as median (interquartile range, IQR) for top walking speed (TWS), and mean (standard deviation, SD) for all other spatiote variables. TWSnorm, normalised top walking speed; POS, posterior approach; DAA, direct anterior approach; CON, control group. Sample sizes**:** n = 17 (DAA); n = 17 (POS); n = 19 (CON)

### Ground reaction force profile

**Fig. 3:**
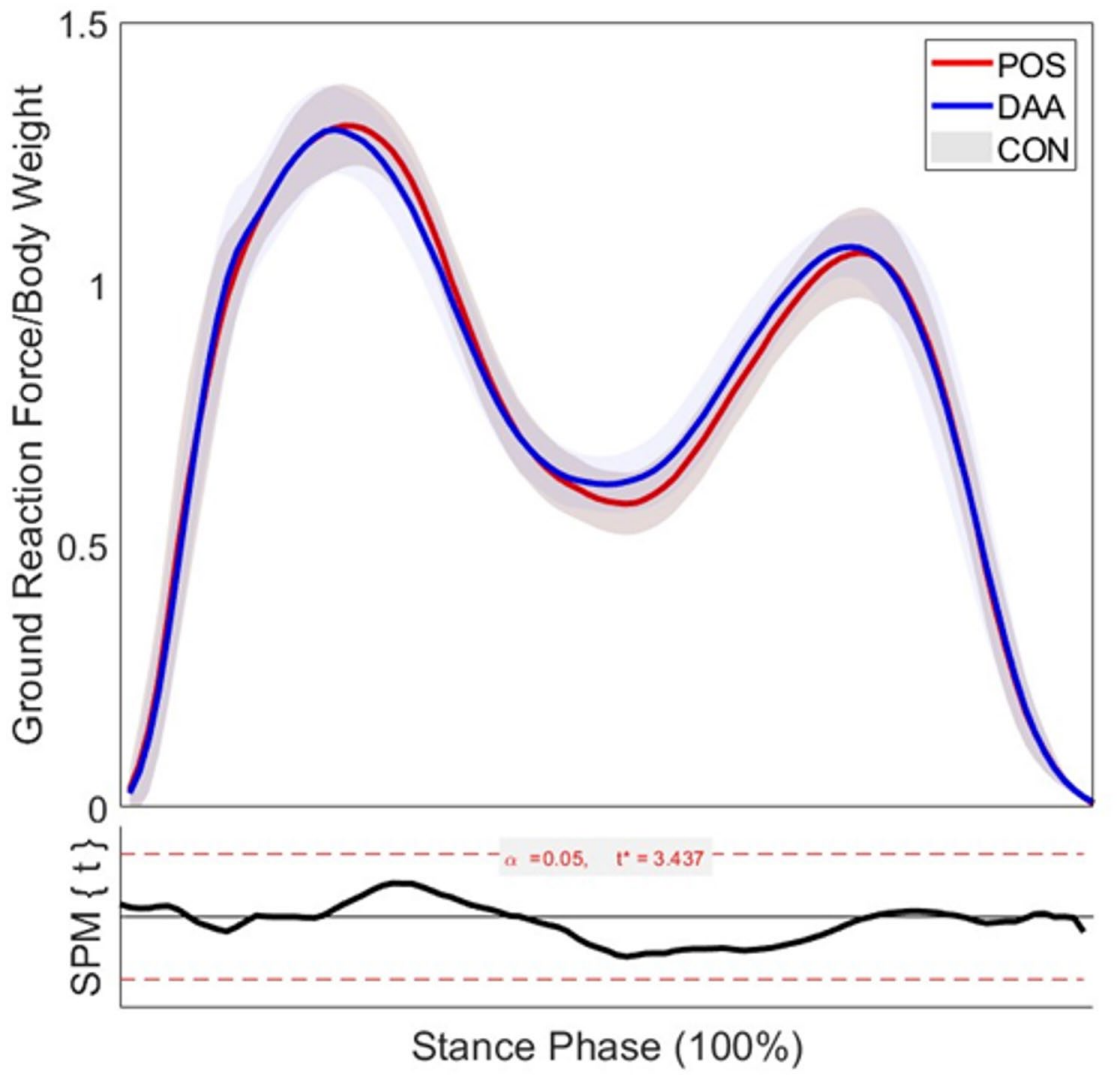
Comparison of vertical ground reaction force during gait following hip resurfacing via anterior and posterior approaches Vertical ground reaction force (vGRF) normalised to body weight across the stance phase of gait in DAA, posterior (POS), and control (CON) groups. All groups exhibit the characteristic biphasic pattern of walking, with no statistically significant differences observed between surgical approaches, as indicated by the Statistical Parametric Mapping (SPM{t}) analysis (bottom panel). Shaded areas represent ± 1 standard deviation. Sample sizes**:** n = 17 (DAA); n = 17 (POS); n = 19 (CON)

SPM analysis detected no significant differences (*p* =  < 0.05) in ground reaction force (GRF) across the stance phase between the POS and DAA groups when compared at 6 km/hr against healthy controls, Fig. 3.

### Hip range of motion

**Fig. 4:**
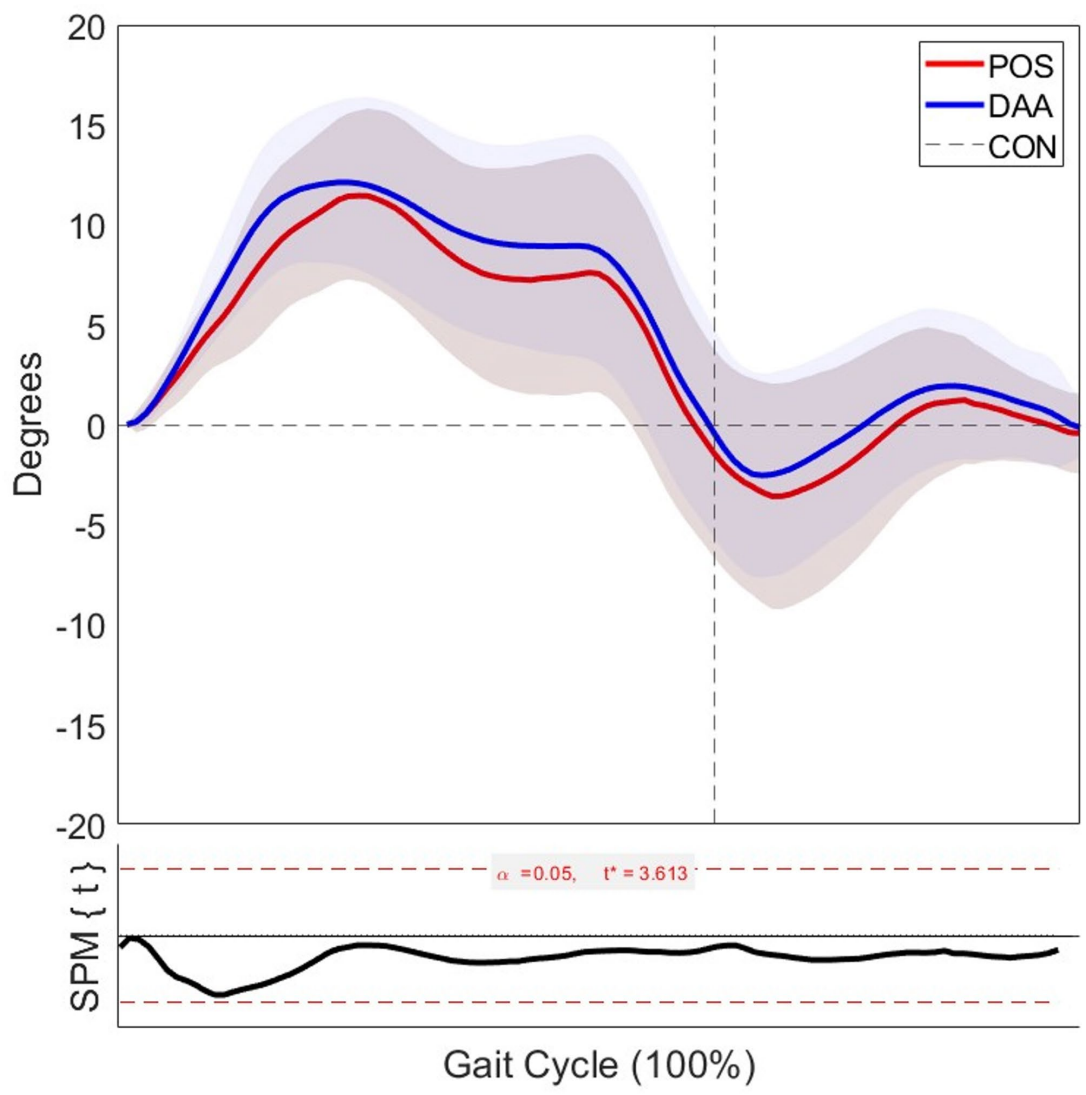
Hip joint range of motion in the coronal plane for the affected leg during gait at 6 km/h, comparing the posterior and direct anterior approaches to healthy controls. Shaded areas represent ± 1 standard deviation. The lower panel displays the SPM{t} statistic comparing POS and DAA across the gait cycle. Sample sizes**:** n = 17 (DAA); n = 17 (POS); n = 19 (CON)

**Fig. 5:**
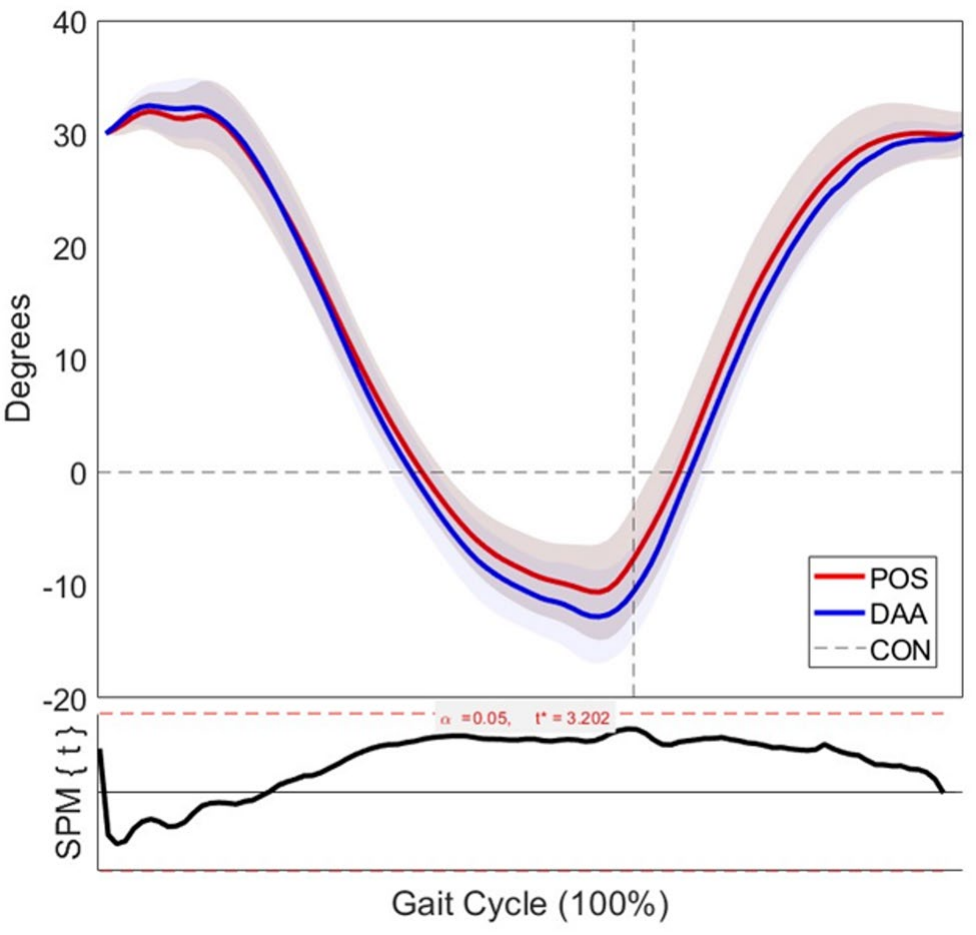
Hip flexion–extension angle across the gait cycle following hip resurfacing via anterior and posterior approaches. Shaded areas represent ± 1 standard deviation. Statistical Parametric Mapping (SPM{t}) analysis is shown in the lower panel. Sample sizes**:** n = 17 (DAA); n = 17 (POS); n = 19 (CON)

Analysis of hip joint kinematics revealed no statistically significant differences in range of motion between POS and DAA approaches across the gait cycle in either the coronal or sagittal planes. In the coronal plane (Fig. 4), both groups exhibited similar patterns of adduction and abduction, with a peak difference of approximately 2 degrees and the SPM{t} statistic remaining below the critical threshold (t* = 3.613, α = 0.05) throughout the gait cycle. In the sagittal plane (Fig. 5), while slight variations were observed—particularly with the DAA group showing marginally greater peak extension—the SPM{t} curve again remained below the critical threshold (t* = 3.202, α = 0.05), indicating no statistically significant difference. Both resurfacing groups demonstrated similar motion profiles overall.

## Discussion

The primary finding is that, at one year postoperatively, patients undergoing HRA via either the direct anterior or posterior approach demonstrate gait patterns comparable to age-matched healthy controls. This is particularly relevant for resurfacing candidates, who are typically higher-demand, as it indicates that surgical approach does not compromise the key advantage of HRA—the restoration of near-native biomechanics. The secondary finding is that HRA through either approach delivers excellent PROMs—maximal OHS, and a MET score averaging 13, which equates to the ability to perform high-intensity activities such as running, vigorous cycling, or competitive sports, reflecting exceptional functional capacity postoperatively.

Our findings align with previous studies analysing spatiotemporal variables, demonstrating that HRA can restore near-physiological gait [9, 10]. Furthermore, this study is the first to assess multiplanar hip angle kinematics using SPM throughout the gait cycle, showing restoration to near-normal levels following resurfacing with either approach. A comparable study [16] investigated the three most commonly utilised surgical approaches to the hip— DAA, POS, and LAT—in the context of total hip arthroplasty. Ryan et al. found that the DAA and lateral approaches resulted in more favourable sagittal and frontal plane gait kinematics compared to the posterior approach. Only the DAA group demonstrated spatiotemporal parameters within the normal range. Notably, their DAA cohort exhibited evidence of hip abductor unloading during the loading response phase—a pattern not observed in our study. At one year, when gait recovery has typically plateaued, our findings suggest that once native anatomy is preserved—as in resurfacing—surgical approach has little effect on biomechanics.

The mechanisms underlying the ability of hip resurfacing to restore more normal gait remain unclear. To date, one randomised controlled trial [8] has described its superiority over THA, particularly at higher walking speeds. However, this was not replicated in other trials [27], with Lavigne et al. [28] concluding that large-head THA provides the same benefit to restoring gait as HRA. Their trial may have been inadequately powered [29]. Biomechanical studies have demonstrated the role of the posterior hip capsule in wrapping around the femoral head in deep flexion [30]. Regardless of surgical approach, restoring native head size by resurfacing the head, and repairing the capsule, restores capsular tension and its wrapping function. Conversely, THA with a 32mm head left the capsule slack thus defunctioning the capsule, and this effect was more pronounced with posterior compared to anterior capsulotomy, regardless of repair. Interestingly, an article comparing gait parameters across different head sizes found that a larger head size (36 mm) improved gait patterns [31] at a mean of 3.5 years postoperatively. Longer-term functional benefits of larger head sizes (36–44mm) have also been described using the UCLA score at five years [32]. However, the clinical translation of the biomechanical evidence for restoring capsular tension has not previously been established for hip resurfacing.

At higher activity levels such as fast walking or running, peak hip stresses can reach seven times body weight [33]. The resulting load transfer to the proximal femur is impacted by the disruption of the trabeculae in the proximal femur delivering load from the major muscle groups [34]. HRA has been shown to preserve native proximal femoral elasticity [35, 36] but the influence of fixation method, femoral stem size, and materials on proximal femoral elasticity, particularly with emerging CoC HRA, remains understudied.

Current clinical evidence indicates that DAA facilitates faster functional recovery, consistent with lower early postoperative pain scores [15]. These advantages have contributed to its widespread adoption, with DAA now the predominant approach in multiple European countries [37–39] and rising in the USA [40]. However, an additional learning curve exists even for experienced DAA surgeons when adapting the approach for resurfacing. Balancing adequate exposure, femoral mobility, and capsular blood supply preservation is achievable but influenced by patient factors [41]. While the posterior approach may provide easier exposure for surgeons trained in the posterior approach, our findings indicate comparable functional outcomes between approaches.

This study has a number of limitations. First, it offers only a snapshot of data at approximately one year postoperatively. This study forms part of a broader investigation of gait outcomes across different arthroplasty types; accordingly, we selected the 12-month postoperative time point, when gait metrics typically stabilise, to enable robust comparisons with healthy controls. The direct anterior approach does appear to provide earlier functional gains and shorter hospital stay for THA [36]— so any short-term differences between approaches may have been missed in this study. Future work could incorporate serial gait assessments at early and later time points or the use of pervasive monitoring to define recovery trajectories and any time-dependent differences between approaches.

Secondly, the lack of pre-operative data, and the fact that these were consecutive not randomised series limits baseline comparisons and precludes assessment of preoperative impairment severity on postoperative outcomes. We are however reassured by the fact our patients reached the levels of healthy controls despite suffering with debilitating osteoarthritis preoperatively. Furthermore, only male patients were included, as female sex is considered a contraindication for MoM HRA. Lastly the two series are sequential, as the senior author changed approach two years after changing to DAA for THA and being convinced that it offered objective benefits for patients. There may therefore be a bias towards worse outcomes in the DAA group, while the POS group are well past any learning curve.

Although the non-randomised design introduces a risk of selection bias, we employed robust demographic matching across the three groups to minimise this risk. This prospective study incorporated multimodal gait analysis (kinematics and kinetics), post-operative hip scores, and personalised physical-activity assessment using the MET score [25].

Objective functional measures, such as gait analysis, are clinically relevant in evaluating arthroplasty outcomes, as limited walking speed often indicates reduced capacity for high-demand physical activities [11] at one extreme, while being related to risk of falls in older patients [42]. As physical function improves, patients may be better able to engage in moderate-to-vigorous physical activity (MVPA), which is strongly associated with reduced cardiometabolic disease risk and lower all-cause mortality [43, 44]. In line with this, current WHO guidelines recommend that adults maintain 150–300 min of moderate-intensity aerobic or at least 75–150 min of vigorous-intensity [45]. This equates to between 450–900 Metabolic Equivalent of Task (MET) minutes. In our study, the high postoperative MET scores for both approaches ( POS 13.1, DAA 12.6) indicating engagement in MVPA, reflecting high functional recovery. Crucially, the MET score mitigated the ceiling effect of the OHS by capturing levels of physical activity that were previously undetectable, as demonstrated in Figs. 1 and 2. These postoperative MET values are comparable to those reported by Edwards et al. [25], who found mean scores of 12–13 following hip arthroplasty, further supporting the validity of our findings. Although preoperative MET data were not available, the postoperative values indicate restoration of physical activity levels typical of healthy adults meeting or exceeding recommended thresholds for vigorous-intensity exercise. In cardiopulmonary literature using treadmills, each 1-MET higher level of cardiorespiratory fitness has been associated with a ~ 12–13% reduction in all-cause mortality [46, 47]. While our MET score represents habitual activity rather than maximal exercise capacity, these findings provide meaningful context, suggesting that hip resurfacing—regardless of approach—restores activity levels consistent with long-term health benefits.

When performed safely, DAA appears to be equally effective for hip resurfacing in high-demand patients, supporting the restoration of function to levels enabling high-intensity health enhancing physical activity. Future studies should incorporate randomisation, preoperative gait analysis, and postoperative activity tracking via accelerometers at multiple time points to determine whether the difference in outcome between approaches is clinically important.

## Conclusions

This study reports equivalent outcomes between the anterior and posterior approaches for HRA at one year postoperatively, with both restoring gait comparable to healthy controls with no statistical differences observed between DAA and POS. Both approaches yielded successful outcomes, suggesting that surgeons should perform hip resurfacing using the approach with which they are most familiar.

## Data Availability

The datasets generated and/or analysed during the current study are available from the corresponding author on reasonable request.

## Abbreviations

DAA: Direct anterior approach
POS: Posterior approach
HRA: Hip resurfacing arthroplasty
THA: Total hip arthroplasty
BHR: Birmingham hip resurfacing
MoM: Metal-on-metal
CoC: Ceramic-on-ceramic
OA: Osteoarthritis
PROMs: Patient-reported outcome measures
OHS: Oxford hip score
EQ-5D: EuroQol 5-dimension
BMI: Body Mass Index
CON: Control group
GRF: Ground reaction force
TWS: Top walking speed
SPM: Statistical parametric mapping
KDE: Kernel density estimation
MVPA: Moderate-to-vigorous physical activity
WHO: World Health Organization
SD: Standard deviation
IQR: Interquartile range
ANOVA: Analysis of variance
MATLAB: Matrix Laboratory (software)
